# Oxidation of KCNB1 potassium channels triggers apoptotic integrin signaling in the brain

**DOI:** 10.1038/cddis.2017.160

**Published:** 2017-04-06

**Authors:** Wei Yu, Manasa Gowda, Yashsavi Sharad, Surindo A Singh, Federico Sesti

**Affiliations:** 1 Department of Neuroscience and Cell Biology, Rutgers University, Robert Wood Johnson Medical School, 683 Hoes Lane West, Piscataway, NJ, USA

## Abstract

Oxidative modification of the voltage-gated potassium (K^+^) channel KCNB1 promotes apoptosis in the neurons of cortex and hippocampus through a signaling pathway mediated by Src tyrosine kinases. How oxidation of the channel is transduced into Src recruitment and activation, however, was not known. Here we show that the apoptotic signal originates from integrins, which form macromolecular complexes with KCNB1 channels. The initial stimulus is transduced to Fyn and possibly other Src family members by focal adhesion kinase (FAK). Thus KCNB1 and integrin alpha chain V (integrin-*α*_5_) coimmunoprecipitated in the mouse brain and these interactions were retained upon channel's oxidation. Pharmacological inhibition of integrin signaling or FAK suppressed apoptosis induced by oxidation of KCNB1, as well as FAK and Src/Fyn activation. Most importantly, the activation of the integrin–FAK–Src/Fyn cascade was negligible in the presence of non-oxidizable C73A KCNB1 mutant channels, even though they normally interacted with integrin-α_5_. This leads us to conclude that the transition between the non-oxidized and oxidized state of KCNB1 activates integrin signaling. KCNB1 oxidation may favor integrin clustering, thereby facilitating the recruitment and activation of FAK and Src/Fyn kinases.

The delayed rectifier, voltage-gated potassium (K^+^) channel KCNB1, carries a major somatodendritic current in the cortex and hippocampus.^1, 2^ Loss-of-function mutations in KCNB1 have been linked to early infantile epileptic encephalopathy^3, 4, 5^ and KCNB1 knock out in mice causes hippocampal hyperexcitability and seizures.^6^ Additionally, during conditions of oxidative stress, reactive oxygen species (ROS) act to modify KCNB1 channels in a manner that they become cytotoxic.^7, 8, 9, 10^ For example, in mouse model of traumatic brain injury (TBI), oxidized KCNB1 channels contribute to tissue damage and consequent behavioral impairment.^9^ Oxidized KCNB1 channels are also present in the brains of aging mice and in larger amounts in the brain of 3x-Tg-AD mouse model of Alzheimer's disease,^8, 11, 12, 13, 14, 15, 16^ where they promote hyperexcitability and presumably apoptosis.^17^ Thus oxidative modification of the KCNB1 channel represents an important mechanism of neuronal vulnerability that has the potential to affect multiple processes in the brain and in the other organs where KCNB1 operates, including the pancreas.^18^ At the molecular level, oxidants crosslink KCNB1 subunits to each other through disulfide bridges involving cys73 in the N-terminus.^8^ These KCNB1 oligomers do not conduct current and are poorly endocytosed.^7^ The accumulation of KCNB1 oligomers in the plasma membrane that follows is associated with the activation of Src tyrosine kinases and downstream C-Jun N-terminal kinases (JNKs), which act to destabilize mitochondria leading to further ROS leakage and finally apoptosis.^7, 9^ The molecular steps that link oxidized KCNB1 channels to downstream recruitment and activation of Src tyrosine kinases are not known and this question is significant, considering the role that oxidation of this channel seems to have for normal aging and disease. Previous studies have established a mechanistic link between KCNB1 and focal adhesion kinase (FAK) in the processes associated with cell motility.^19, 20^ Thus Wei *et al.*^19^ showed that KCNB1 can induce FAK autophosphorylation at tyr397 and in this manner control cell polarization and migration. It is well established that autophosphorylation of FAK at tyr397 promotes its association and activation of Src kinases,^21^ which also have a key role in the apoptotic pathway activated by oxidation of KCNB1. This evidence, along with the fact that the FAK-activating signal originates in the first 50 amino acids of the N-terminus of KCNB1,^19^ a region not distant from cys73, led us to speculate that FAK signaling might have a role in the apoptotic mechanisms associated with oxidation of the channel. Here we show that KCNB1 and integrins form stable complexes in the membranes of neurons of the mouse brain and of mammalian cells. The changes that occur when the channel transitions from a non-oxidized to an oxidized state stimulate integrin signaling leading to autophosphorylation of FAK at tyr397 and consequent activation of Fyn, a member of the SrcA sub-family.

## Results

### Integrins form stable complexes with KCNB1 channels in CHO cells and brain

**Figure 1:**
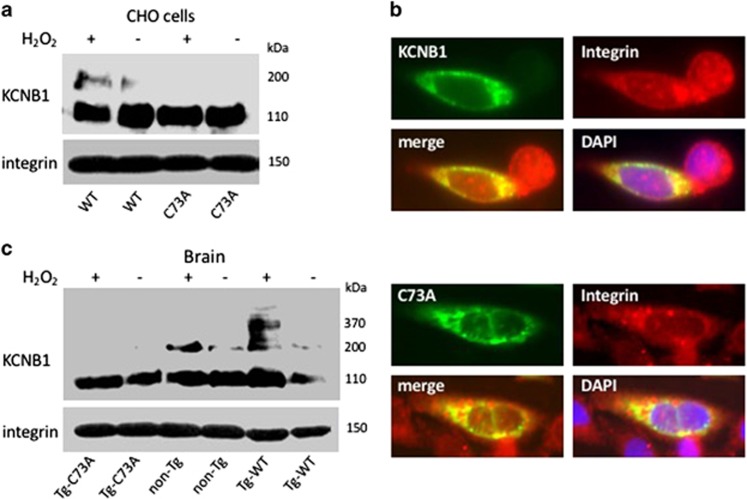
Integrins coimmunoprecipitate with KCNB1 channels. (**a**) CHO cells were transfected with cDNA encoding human WT or C73A HA-tagged KCNB1 subunits and lysed in control conditions or subjected to an oxidative insult (1.0 mM H_2_O_2_ for 5 min), prior to lysis. In the upper immunoblot, integrin-*α*_5_ immunoprecipitates were visualized with anti-HA antibodies to detect KCNB1 protein. Total lysates (lower blot) were stained with anti-integrin-α_5_. Integrins were detected in a single ∼150 kDa band. (**b**) Representative images of CHO cells demonstrating colocalization of WT and C73A with integrins. The cells were transfected with pEGFP-N1-WT or pEGFP-N1-C73A and stained with integrin primary antibody and rhodamine-conjugated secondary antibody. Images were analyzed using the ImageJ software. (**c**) Brain lysates from the indicated genotypes were treated in control conditions or in the presence of 1.0 mM H_2_O_2_ for 5 min, prior to the addition of sample buffer. In the representative immunoblots, integrin-α_5_ immunoprecipitates were visualized with an antibody against mouse and human KCNB1. Total lysates were stained with anti-integrin-α_5_

To determine whether integrins and KCNB1 channels can interact and whether these associations are retained following oxidation of the channel, we carried out coimmunoprecipitation experiments in Chinese hamster ovary (CHO) cells transiently expressing human KCNB1 epitope tagged to the HA tag in the C-terminus (we showed previously that the addition of the tag has no effect on the properties of the channel^8^). KCNB1 was oxidized by exposing the cells to 1.0 mM hydrogen peroxide, (H_2_O_2_) for 5 min prior to lysis. Proteins were immunoprecipitated (IP) with an antibody that detects integrins alpha chain V (integrin-α5) and immunoblotted with a HA antibody to detect KCNB1 protein. As a control, we used a KCNB1 mutant, C73A, which does not oligomerize.^8^ Representative western blots of six coimmunoprecipitation experiments are shown in Figure 1. The lower blot in Figure 1a shows staining of total lysates with integrin-α5 antibody. Coimmunoprecipitations are shown in the upper blot in the figure. Oxidized KCNB1 channels in CHO cells or in the mouse brain form oligomers that can be detected in multiple bands ranging from ∼170 to ∼400 kDa.^8^ Indeed, integrin-α5 pulled down both the non-oxidized (∼110 kDa) and oxidized (∼200 kDa) forms of wild-type (WT) KCNB1 indicating that the channel interacted with endogenous integrins in CHO cells and that these interactions were retained upon its oxidation/oligomerization. Also C73A channels formed a complex with the integrins but in this case only the non-oxidized band was detected in the blot because C73A does not oligomerize.

### KCNB1 and integrins colocalize in CHO cells

To further strengthen the notion that KCNB1 channels and integrins form a complex, we assessed their surface colocalization in CHO cells. For these studies, we employed a green fluorescent protein (GFP)-KCNB1 fusion in the N-terminus (pEGFP-N1-WT or pEGFP-N1-C73A), stained CHO cells with integrin-α5 antibody and imaged immunofluorescence using Nomarski microscopy. In agreement with coimmunoprecipitation results, strong colocalization of KCNB1 and integrins was detected on the surface of CHO cells transfected with either pEGFP-N1-WT or pEGFP-N1-C73A (Figure 1b). The lack of strong colocalization in the cells' cytoplasm may indicate that KCNB1 and integrins traffic to the plasma membrane following different routes.

### KCNB1 and integrins form stable complexes in the mouse brain

We next determined whether KCNB1 formed a complex with integrins in the mouse brain. Integrin-α5 were IP from the lysates of half-brains of 3-month-old mice treated in the absence/presence of 1.0 mM H_2_O_2_ for 5 min before the addition of sample buffer and immunoblotted with a KCNB1 antibody that detects a C-terminal epitope conserved in the mouse and human channel. To ascertain whether integrins interacted with C73A mutant channels in the brain, we took advantage of transgenic mice expressing human C73A tagged to the HA tag in the C-terminus (Tg-C73A) in the cortex and hippocampus and, as an additional control, of transgenic mice expressing WT tagged to the HA tag in the C-terminus (Tg-WT) that we previously characterized.^9^ In those animals, exogenous and endogenous KCNB1 subunits appear to form heteromeric complexes (indeed, mouse and human KCNB1 share 97% amino-acid sequence identity^9^). As a result, the amounts of oxidized KCNB1 channels are small in the Tg-C73A brain, because the cys73 to ala mutation is dominant negative while in the Tg-WT brain due to overexpression they are larger than in non-Tg.^9^ Thus, in agreement with results obtained using CHO cells, integrins-α5 formed stable complexes with KCNB1 channels and these interactions were maintained during channels' oligomerization (∼200–∼370 kDa bands, Figure 1c). Similar interactions were detected in the Tg-WT and Tg-C73A brains, as expected.

Taken together, these results lead us to conclude that integrins are general partners of KCNB1 channels.

### Inhibition of integrins prevents KCNB1-induced apoptosis

**Figure 2:**
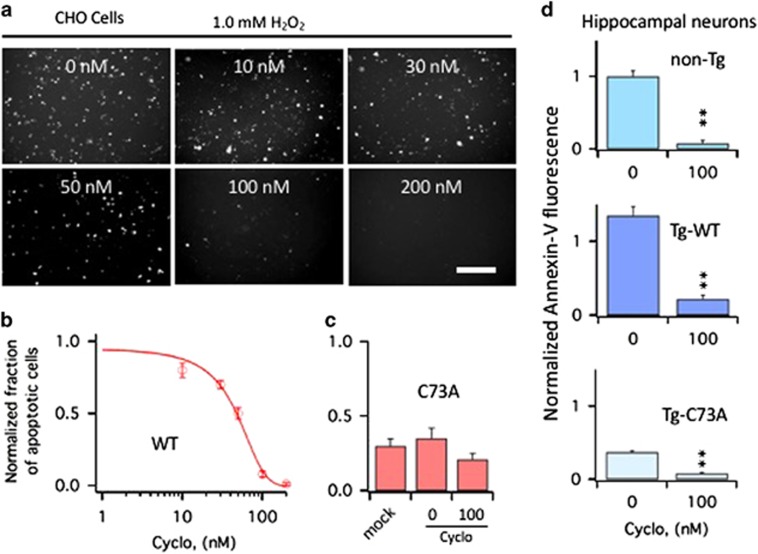
Cyclo protects CHO cells and primary hippocampal neurons from apoptosis induced by oxidation of KCNB1. (**a**) Representative examples of Annexin-V staining in CHO cells transfected with WT. The cells were exposed to 1.0 mM H_2_O_2_ for 5 min and then incubated 6 h in the presence of Cyclo at the indicated nanomolar concentrations. At the end of the 6-h incubation period, cells were stained with Annexin-V and photographed. Size bar 100 *μ*m. (**b**) Number of WT-transfected CHO cells subjected to an oxidative challenge positive to Annexin-V in the absence/presence of varying concentrations of Cyclo. Data are normalized to the number of cells positive to Annexin-V in the absence of Cyclo. Data were fit to a sigmoidal function (equation (1)) with an IC_50_=41.4 nM. *N*=3 experiments. (**c**) Number of mock or C73A-transfected CHO cells positive to Annexin-V subjected to an oxidative challenge in the absence or presence of 100 nM Cyclo. *N*=3 experiments. (**d**) Quantitative assessment of Annexin-V fluorescence (proportional to the number of cells undergoing apoptosis) in primary hippocampal neurons of the indicated genotypes subjected to an oxidative challenge. After wash out, cultures were incubated in the absence/presence of 100 nM Cyclo for 6 h and then stained with Annexin-V. Fluorescence was assessed using the ImageJ software and data are normalized to non-Tg neurons in the absence of Cyclo. *P*=1.8 × 10^−10^ (one-way analysis of variance), **P<0.01 (Tukey's *post hoc*). *N*=3 embryos/genotype

The finding that KCNB1 interacts with integrins led us to speculate that the oligomerization of the channel that follows its oxidation might activate integrin signaling, for example, by stimulating their clustering, which drives the recruitment and activation of Src kinases via FAK.^22, 23^ If integrins are responsible for the apoptotic stimulus, pharmacologically inhibiting integrin signaling should prevent apoptosis induced by oxidation of KCNB1, whereas inhibiting integrin signaling in cells expressing C73A should have only minor effect on their viability. To test this hypothesis, cells were subjected to an oxidative insult and then incubated in the presence of integrin inhibitor Cyclo (-RGDfK) or vehicle. Early apoptosis was assessed 6 h postoxidation by Annexin-V staining, as carried out before.^7, 8, 9^ Thus cells expressing WT were susceptible to apoptosis in agreement with previous studies^8^ (Figures 2a and b). Cells expressing the C73A mutant exhibited a significantly lower rate of apoptotic death than cells transfected with WT; in fact, apoptosis levels in C73A-transfected cells were comparable to those of mock-transfected cells (Figure 2c). Cyclo inhibited apoptosis in CHO cells expressing the WT channel in a dose-dependent manner, with an IC_50_=38.6±2.3 nM (Figure 2b). Also primary hippocampal neurons obtained from non-Tg and Tg-WT embryos were susceptible to oxidant-induced apoptosis (Figure 2d. For representative images, see Figure 4). In agreement with previous studies,^9^ Tg-C73A neurons retained moderated apoptosis susceptibility probably due to the residual oxidation of the channel in the brain of this genotype. When the cultures were treated with Cyclo, the inhibitor significantly suppressed apoptosis in all genotypes.

### Integrins stimulate FAK autophosphorylation at tyr397

**Figure 3:**
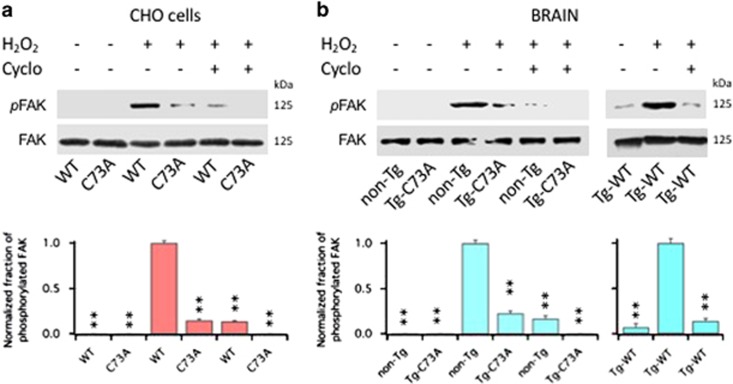
Cyclo inhibits FAK activation induced by oxidation of KCNB1. (**a**) Representative western blots showing autophosphorylated FAK at tyr397 (pFAK) and total FAK (FAK) in CHO cells transfected with WT or C73A in the absence/presence of 200 nM Cyclo. FAK protein was detected into a single, ∼125 kDa band. Cells were oxidized with 1.0 mM H_2_O_2_ for 5 min and then incubated in control media or in media containing 200 nM Cyclo for 1 h before lysis. Quantifications of three experiments are shown in the lower panel and are normalized to WT+H_2_O_2_. *P*=1.0 × 10^−8^ (one way analysis of variance (ANOVA)). **P<0.01 for pairwise comparisons *versus* WT+H_2_O_2_ (Tukey's *post hoc* test). (**b**) Representative western blots showing phosphorylated FAK (pFAK) and total FAK (FAK) in the brains of the indicated genotypes in the absence/presence of 200 nM Cyclo. Brain lysates were incubated 1 h in the absence/presence of 1.0 mM H_2_O_2_ and the absence/presence of 200 nM Cyclo. The reactions were stopped by adding sample buffer to the lysates. For non-Tg and Tg-C73A, quantifications of three experiments are shown in the lower panel and are normalized to non-Tg+H_2_O_2_. *P*=1.4 × 10^−6^ (one-way ANOVA). **P<0.01 for pairwise comparisons *versus* non-Tg+H_2_O_2_ (Tukey's *post hoc* test). For Tg-WT, quantifications of two experiments are shown in the lower panel and are normalized to Tg-WT+H_2_O_2_. *P*=1.7 × 10^−4^ (one-way ANOVA). **P<0.01 for pairwise comparisons *versus* Tg-WT+H_2_O_2_ (Tukey's *post hoc* test)

To determine whether oxidation of KCNB1 could induce activation of FAK via integrin signaling, we assessed the fraction of autophosphorylated FAK at tyr397 in CHO cells transfected with WT or C73A. Representative western blots of these experiments along with quantitative analyses are shown in Figure 3a. Thus autophosphorylated FAK proteins were detected in WT-expressing cells that had been exposed to an oxidative insult and in significantly smaller amounts, in cells expressing C73A (Figure 3a). Most importantly, FAK autophosphorylation was markedly reduced in cells exposed to 200 nM Cyclo. *Ex vivo* experiments with mouse brains, shown in Figure 3b, recapitulated results obtained with CHO cells (brain homogenates were preferred over primary neurons because the brain provides a more physiological system). Thus autophosphorylated FAKs were detected in lysates of non-Tg brains and Tg-WT brains exposed to an oxidative insult, whereas their amounts were lower in lysates of Tg-C73A brains. In all cases, 200 nM Cyclo significantly decreased FAK autophosphorylation at tyr397. The amounts of autophosphorylated FAK were ∼36% larger in the Tg-WT brains compared with the non-Tg brains, consistent with the presence of higher amounts of oxidized KCNB1 channels in the former (in the figure, data are plotted normalized to their internal control).

### Inhibition of FAK protects against KCNB1-mediated apoptosis

**Figure 4:**
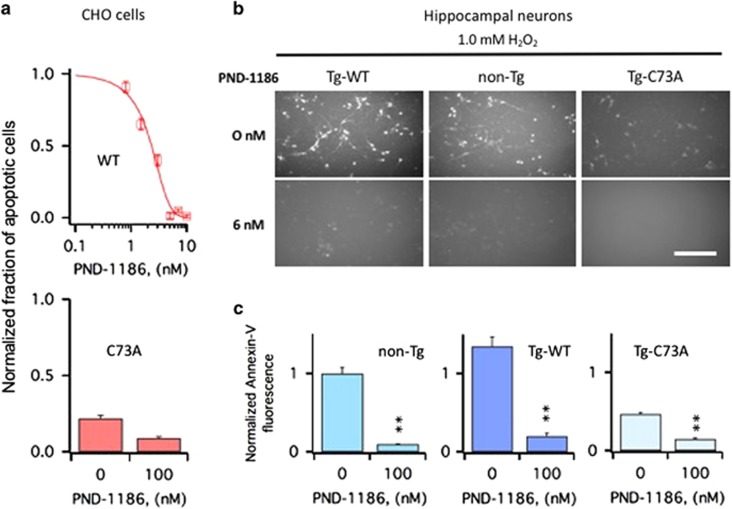
PND-1186 protects CHO cells and primary hippocampal neurons from apoptosis induced by oxidation of KCNB1. (**a**) Upper panel: number of cells positive to Annexin-V in WT-transfected CHO cells subjected to an oxidative challenge in the absence/presence of varying concentrations of PND-1186. Data are normalized to the number of cells positive to Annexin-V in the absence of PND-1186. Data were fit to a sigmoidal function (equation (1)) with an IC_50_=1.95 nM. *N*=3 experiments. Lower panel: number of C73A-transfected CHO cells positive to Annexin-V subjected to an oxidative challenge in the absence or presence of 6 nM PND-1186. *N*=3 experiments. (**b**) Representative examples of Annexin-V staining in the primary hippocampal neurons of the indicated genotypes. The cells were exposed to 1.0 mM H_2_O_2_ for 5 min, incubated 6 h in the presence of 6 nM PND-1186, stained with Annexin-V and photographed. Size bar 100 *μ*m. (**c**) Quantitative assessment of Annexin-V fluorescence in the primary hippocampal neurons of the indicated genotypes subjected to an oxidative challenge in the absence/presence of 6 nM PND-1186. Annexin-V fluorescence was calculated using the ImageJ software and data are normalized to non-Tg neurons in the absence of PND-1186. *P*=3.9 × 10^−7^ (one-way analysis of variance); **P<0.01 (Tukey's *post hoc*). *N*=3 embryos/genotype

Annexin-V staining following an oxidative insult was significantly lowered in CHO cells expressing WT treated with FAK inhibitor PND-1186 (Figure 4a). The effect of the inhibitor was dose dependent with an IC_50_=2.1±0.1 nM. In contrast, the inhibitor had only a marginal effect in cells transfected with C73A, which are characterized by low levels of apoptosis. The antiapoptotic effect of PND-1186 was further assessed in primary hippocampal neurons subjected to an oxidative insult (Figures 4b and c). Representative images of Annexin-V staining in the cultures of hippocampal neurons are shown in Figure 4b. Quantification of those experiments, shown in Figure 4c, confirmed that PND-1186 significantly inhibited apoptosis in all genotypes.

### Integrins activate Fyn tyrosine kinases

**Figure 5:**
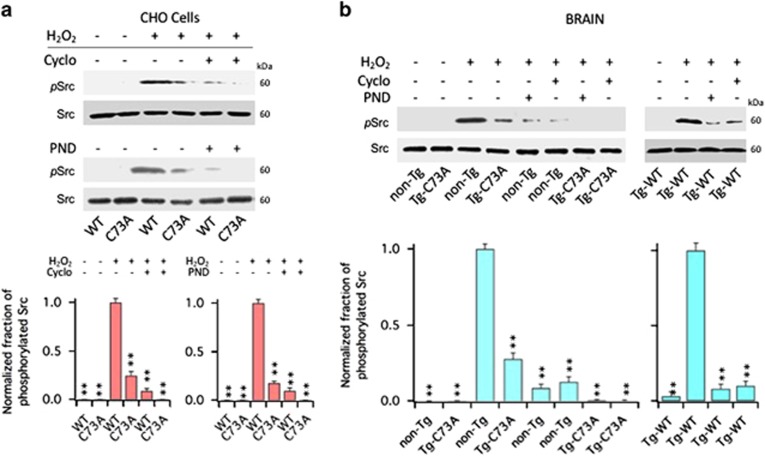
Cyclo and PND-1186 inhibit Src activation induced by oxidation of KCNB1. (**a**) Representative western blots showing phosphorylated Src at Tyr 416 (pSrc) and total Src (Src) in CHO cells transfected with WT or C73A in the absence/presence of the indicated inhibitors. Src protein was detected into a single, ∼60 kDa band. Cells were oxidized with 1.0 mM H_2_O_2_ for 5 min and then incubated in control media or in media containing 200 nM Cyclo or 10 nM PND-1186 (PND) for 1 h before lysis. Quantifications of three experiments are shown in the lower panel and are normalized to WT+H_2_O_2_. *P*=2.9 × 10^−6^ and 3.5 × 10^−6^ for Cyclo and PND-1186, respectively (one-way analysis of variance (ANOVA)). **P<0.01 for pairwise comparisons *versus* WT+H_2_O_2_ (Tukey's *post hoc* test). (**b**) Representative western blots showing phosphorylated Src (pSrc) and total Src (Src) in the brains of the indicated genotypes in the absence/presence of the indicated inhibitors. Brain lysates were incubated 1 h in the absence/presence of 1.0 mM H_2_O_2_ and the absence/presence of 200 nM Cyclo or 10 nM PND-1186 (PND). The reactions were stopped by adding sample buffer to the lysates. Quantifications of three experiments with non-Tg and Tg-C73A are shown in the lower panel and are normalized to non-Tg+H_2_O_2_. *P*=5.3 × 10^−8^ (one-way ANOVA). **P<0.01 for pairwise comparisons *versus* non-Tg+H_2_O_2_ (Tukey's *post hoc* test). Quantifications of two experiments with Tg-WT are shown in the lower panel and are normalized to Tg-WT+H_2_O_2_. *P*=1.9 × 10^−7^ (one-way ANOVA). **P<0.01 for pairwise comparisons *versus* Tg-WT+H_2_O_2_ (Tukey's *post hoc* test)

**Figure 6:**
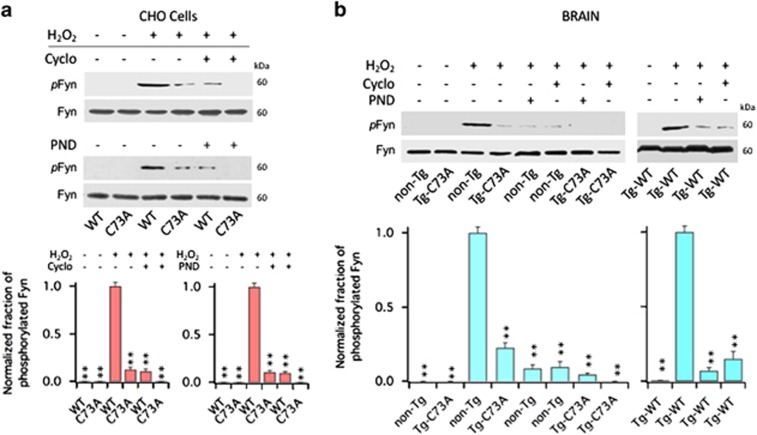
Cyclo and PND-1186 inhibit Fyn activation induced by oxidation of KCNB1. (**a**) Representative western blots showing phosphorylated Fyn at tyr530 (pFyn) and total Fyn (Fyn) in CHO cells transfected with WT or C73A in the absence/presence of the indicated inhibitors. Fyn protein was detected into a single, ∼60 kDa band. Cells were oxidized with 1.0 mM H_2_O_2_ for 5 min and then incubated in control media or in media containing 200 nM Cyclo or 10 nM PND-1186 (PND) for 1 h before lysis. Quantifications of three experiments are shown in the lower panel and are normalized to WT+H_2_O_2_. *P*=1.3 × 10^−6^ and 7.7 × 10^−7^ for Cyclo and PND-1186, respectively (one-way analysis of variance (ANOVA)). **P<0.01 for pairwise comparisons *versus* WT+H_2_O_2_ (Tukey's *post hoc* test). (**b**) Representative western blots showing phosphorylated Fyn (pSrc) and total Fyn (Fyn) in the brains of the indicated genotypes in the absence/presence of Cyclo or PND-1186. Brain lysates were incubated 1 h in the absence/presence of 1.0 mM H_2_O_2_ and the absence/presence of 200 nM Cyclo or 10 nM PND-1186 (PND). The reactions were stopped by adding sample buffer to the lysates. Quantifications of three experiments with non-Tg and Tg-C73A are shown in the lower panel and are normalized to non-Tg+H_2_O_2_. *P*=9.8 × 10^−8^ (one-way ANOVA). **P<0.01 for pairwise comparisons *versus* non-Tg+H_2_O_2_ (Tukey's *post hoc* test). Quantifications of two experiments with Tg-WT are shown in the lower panel and are normalized to Tg-WT+H_2_O_2_. *P*=1.5 × 10^−4^ (one-way ANOVA). **P<0.01 for pairwise comparisons *versus* Tg-WT+H_2_O_2_ (Tukey's *post hoc* test)

We next determined whether FAK was responsible for the recruitment and activation of Src kinases that follows oxidation of KCNB1. To asses the fraction of activated Src kinases, we used an antibody that detects phosphorylation status of tyr416, a residue conserved in all members of the Src kinase family, as carried out before.^9, 24^ Thus, in CHO cells transfected with WT or C73A, Src phosphorylation at tyr416 was negligible at baseline (Figure 5a). Following an oxidative insult, Src phosphorylation was significantly increased in cells expressing the WT channel compared with cells expressing the C73A mutant, and most importantly, treatments with 200 nM Cyclo or 10 nM PDN-1186 suppressed it. Similar results were observed in the brains of non-Tg, Tg-WT and Tg-C73A mice, with the amounts of phosphorylated Src ∼32% larger in the Tg-WT brains compared with the non-Tg brains (Figure 5b. In the figure, data are plotted normalized to their internal control). The family of Src tyrosine kinases is composed of nine members, including Fyn, which belongs to the SercA sub-family and has been implicated in TBI and Alzheimer's disease, two conditions associated with robust KCNB1 oxidation.^8, 9, 17, 25, 26^ Therefore, we next sought to determine whether oxidation of KCNB1 resulted in the activation of Fyn using a specific antibody that recognizes phosphorylated tyr530 in Fyn. Figure 6a shows the fraction of phosphorylated Fyn kinases in CHO cells transfected with WT or C73A and Figure 6b in the brain lysates of non-Tg, Tg-WT and Tg-C73A in control conditions or following exposure to 1.0 mM H_2_O_2_. The fraction of phosphorylated Fyn protein at tyr530 following an oxidative challenge was significantly increased in all cells expressing the WT channel compared with control and remained low in cells expressing the C73A mutant. Further, Fyn phosphorylation was significantly decreased by treatment with Cyclo or PND-1186.

Together these data led us to conclude that KCNB1 oligomers activate an integrin-FAK-Src/Fyn apoptotic pathway that is conserved in both primary hippocampal neurons and mammalian cells.

## Discussion

In this study, we investigated how oxidation of KCNB1 in neurons of the brain is transduced into an apoptotic signal for Src tyrosine kinases. We found that the apoptotic stimulus originates from integrins, which form macromolecular complexes with KCNB1 channels. Thus KCNB1 and integrin-α_5_ coimmunoprecipitated in the mouse brain and in CHO cells, and most importantly, these interactions were retained upon channel's oligomerization. Moreover, pharmacological inhibition of integrin signaling in primary neurons and CHO cells was protective against apoptosis induced by oxidation of KCNB1. Integrins are heterodimers formed by an *α* and a *β* subunit. We did not attempt to identify which *β*-subunit complements integrin-*α*_5_ in forming a complex with KCNB1, but it is possible that large heterogeneity may exist. For example, integrin-*α*_5_ can form at least four distinct heterodimers with subunits *β*_1_, *β*_5_, *β*_6_ and *β*_8_ in cortical neurons.^27^

Integrins were found to signal through FAK kinases, which have been previously shown to work in concert with KCNB1 in pathological mechanisms of cell adhesion and migration.^19^ Accordingly, biochemical evidence indicated that oxidation of WT channels was associated with significant FAK and Src phosphorylation, which could be suppressed by pharmacological inhibition of integrin signaling. Further, inhibiting the catalytic activity of FAK diminished apoptosis in primary hippocampal neurons and CHO cells. Integrins activate FAK kinases by stimulating autophosphorylation at Y397.^23^ This promotes the formation of a FAK–Src signaling complex through the creation of a high-affinity binding site for the Src-homology 2 (SH2) domain that leads to maximal FAK catalytic activity.^28^ It is not known at this stage whether Src kinases exert their pro-apoptotic action when they are associated with FAK or alone, but in both cases they have broad signaling capabilities. The Src family is composed of nine members and it is therefore possible that multiple kinases are activated by FAK. We found that Fyn, which belongs to the SercA sub-family, has an active role in the pathway under study. The involvement of Fyn is consistent with the prominent role of this kinase in TBI and Alzheimer's disease, two conditions associated with robust KCNB1 oxidation.^8, 9, 17, 25, 26^ The amounts of phosphorylated Fyn and Src were comparable, but the proteins were detected by different antibodies and therefore the involvement of other Src family members in the apoptotic pathway activated by oxidation of KCNB1 cannot be ruled out.

Activation of the integrin–FAK–Src cascade was modest in the presence of C73A channels even though they normally interacted with integrin-α_5_. The only known difference between WT and C73A channels is that oligomerization is negligible in the latter. This leads us to conclude that the transition between the non-oxidized and oxidized state of KCNB1 activates integrin signaling. KCNB1 oligomers are poorly internalized and tend to build up in the membrane.^7^ Thus oligomerization might favor integrin clustering, thereby facilitating the recruitment and activation of FAK and Src kinases.^22, 23^ In the Tg-C73A brains, the exogenous C73A-HA subunits form heteromeric complexes with endogenous KCNB1 subunits. The C73A mutation is dominant negative so that KCNB1 oligomerization in markedly reduced in the Tg-C73A brains.^9^ The remaining activity of integrin signaling in the presence of C73A may therefore be due to residual oligomerization, or alternatively, KCNB1-independent mechanisms. Future studies will address this important point.

KCNB1-induced phosphorylation of Src kinases leads to the recruitment/activation of JNK kinases, which cause oxidative stress and apoptosis by presumably targeting mitochondria.^7^ However, also KCNB1 channels provide a substrate for Src kinases.^29^ The zinc and calcium ions that are released into the cell's cytoplasm in conditions of oxidative stress trigger an apoptotic insertion of KCNB1 channels into the plasma membrane via phosphorylation of tyr124 by Src kinases and of ser800 by p38 mitogen-activated protein kinases.^29, 30^ Modulation of KCNB1 by p38 kinases has been shown to have a role in conditions, such as hepatitis C and cancer.^31, 32, 33^ Evidence indicates that phosphorylation of tyr124 facilitates phosphorylation of ser800 and further that phosphorylation of the latter is decreased in C73A channels without affecting tyr124.^30^ Even though the oligomerization and insertion mechanisms are thought to be uncoupled and to be active during distinct phases of the apoptotic process,^18^ it is possible that integrins may indirectly affect the phosphorylation of KCNB1 by p38 in pathological conditions through their modulation of Src signaling.

A large body of evidence indicates that many types of K^+^ channels, including Ca^2+^-activated K^+^ (KCa) channels,^34^ G-protein-coupled inwardly rectifying K^+^ (GIRK) channels,^35^ human ether-a-go-go-related gene (HERG) K^+^ channels^36^ and voltage-gated 1.3 K^+^ (Kv1.3) channels,^37^ form macromolecular complexes with integrins. These interactions have important roles for channel localization and regulation. Also KCNB1-FAK signaling – presumably through integrins – is involved in cell adhesion and migration in pathological conditions such as wound repair^19^ and migration of bone marrow mesenchymal stem cells to the site of the injury.^20^ Although the studies presented here add additional support to the notion that integrins are general partners of K^+^ channels, they also reveal an unexpected ‘antagonistic pleiotropic' nature^38^ of those interactions that appear to be beneficial under certain pathophysiological conditions and deleterious under other conditions, such as TBI and Alzheimer's disease.^9, 17^ Integrins have many important signaling functions that range from the regulation of cell growth to apoptosis^22^ and the family of Src tyrosine kinases is a hub for many signal transduction pathways. The mechanisms that determine a specific outcome of the integrin/FAK/Src signaling are poorly understood and await further investigation.

## Conclusions

The major conclusions of this study are:

- KCNB1 channels form macromolecular complexes with integrins. These complexes are detected in neurons of the brain and in CHO cells heterologously expressing KCNB1. Thus the ability to assemble with integrins appears to be an intrinsic property of KCNB1 channels.
- FAK kinases transduce the apoptotic signal of integrins, which lack catalytic activity. It seems plausible that KCNB1 oligomerization promotes integrin clustering, an obligated step toward recruitment of FAK.
- The activation of FAK leads to the recruitment/activation of Src tyrosine kinases and, in particular, of Fyn. However, it is possible that other members of the Src family may contribute to the same signaling pathway.
- These studies underscore a non-conducting function of a K^+^ channel that may have implications for a series of conditions ranging from normal aging to neurodegenerative disease.

## Material and methods

### Reagents

Anti-Src (clone 2108), anti-pSrc at tyr416 (clone 2101), anti-FAK (clone 3285) and anti-pFAK at tyr397 (clone 3283) were purchased from Cell Signaling Technology (Danvers, MA, USA). Anti-Fyn (clone MA1-19331) and anti-pFyn at tyr530 (clone PA5-38115) were purchased from Thermo Fisher (Waltham, MA, USA). Anti-HA (clone H6908) was purchased form Sigma-Aldrich (St. Louis, MO, USA). Anti-Kv2.1 (clone K89/34) was purchased from NeuroMab (University of California Davis), UC Davis (Davis, CA, USA)/NIH (Bethesda, MA, USA). Anti-Integrin-*α*5 (clone sc-10729) and goat anti-rabbit IgG-R (clone sc-2091) were purchased from Santa Cruz biotechnology (Dallas, TX, USA). Cyclo (-RGDfK) dissolved in water (100 mM) and PND-1186 dissolved in dimethyl sulfoxide (DMSO) (100 mM) were purchased from APExBIO (Houston, TX, USA).

### Biochemistry and immunohistochemistry

The detailed procedures are previously described.^9, 39^

#### *In vitro* coimmunoprecipitations

CHO cells (CHO-K1) were grown in DMEM medium and transfected in 6-cm dishes with Lipofectamine 2000 (Invitrogen, Carlsbad, CA, USA). For each transfection, 20 *μ*l of transfection reagent and a total of 4 *μ*g of plasmid DNA were used. Twenty-four hours following transfection, cells were lysed with 1 ml of RIPA buffer (10 mM Tris-Cl pH 8.0, 140 mM NaCl, 1 mM EDTA, 1% Triton X-100, 0.1% sodium deoxycholate, 0.1% SDS, 1 mM PMSF) and protease cocktail inhibitors (Sigma-Aldrich). Cell lysates were centrifuged for 20 min at 4 °C and the supernatant was mixed with 2 *μ*g of integrin-α5 antibody at 4 °C overnight. Then protein A/G agarose beads (Santa Cruz Biotechnology; 30 *μ*l of 50% bead slurry) were added and incubated for 2 h at 4 °C. Samples were washed with RIPA buffer five times and the bound proteins were recovered incubating in SDS sample buffer at 100 °C for 5 min. Proteins were resolved by 8% SDS-PAGE and transferred onto a PVDF membrane. After blocking in a 5% solution of non-fat milk in 0.1% Tween 20–phosphate-buffered saline (PBS) (PBST), membranes were probed with HA antibody or Kv2.1 antibody conjugated to HRP, washed with PBST and incubated with POD chemiluminescence substrates (Roche, Branchburg, NJ, USA) and exposed.

#### *In vivo* coimmunoprecipitations

All experimental protocols involving animals were approved by the Rutgers University IACUC Committee. Frozen, half sagittal brains were homogenized with a glass tissue grinder in lysis buffer (0.32 M sucrose, 5 mM Tris-Cl pH 6.8, 0.5 mM EDTA, 1 mM PMSF) and protease inhibitor cocktail. Samples were centrifuged at 2000 r.p.m. for 10 min and the supernatant was used for biochemical analysis. Protein content was quantified with the Bradford colorimetric assay (Sigma). One milligram of brain lysates were incubated at 4 °C overnight with 2 *μ*g of integrin-α5 antibody. Then protein A/G agarose beads (30 *μ*l of 50% bead slurry) were added and incubated for 2 h at 4 °C. Samples were centrifuged for 30 s at 4 °C, and the pellet was washed five times in cell lysis buffer. The pellet was resuspended with 50 *μ*l 2 × SDS Laemmli buffer, heated at 100 °C for 10 min and centrifuged for 1 min at 14 000 × *g*. The sample was loaded on 8% SDS-PAGE gel and immunoblotted with either HA or Kv2.1 antibody.

#### Colocalization

For colocalization experiments, the human KCNB1 cDNA was subcloned into the pEGFP-N1 vector (Clontech-Takara, Mountain View, CA, USA). Thus CHO cells were transiently transfected with pEGFP-N1-WT or pEGFP-N1-C73A by Lipofectamine 2000 as described. Twenty-four hours later, cells were washed once with PBS and fixed with paraformaldehyde (4% in PBS) for 15 min at room temperature. After being fixed, cells were washed three times for 5 min with PBS and blocked for 1 h at room temperature with 5% normal goat serum in PBS plus 0.3% Triton X-100. Cells were incubated with integrin-α_5_ antibody overnight at 4 °C. After washing three times for 5 min with PBS, cells were incubated with goat anti-rabbit IgG-R (Santa Cruz Biotechnology, sc-2091) for 1 h at room temperature. After being washed three times for 5 min with PBS, slides were mounted in Vectashield Antifade Mounting Medium with DAPI mounting buffer (Vector Laboratories, Burlingame, CA, USA) and stored at 4 °C. Staining was visualized with a Zeiss Axiophot microscope (Thornwood, NY, USA).

Biochemical experiments were analyzed using the ImageJ software (NIH).

### Preparation of hippocampal neuronal cultures

The detailed procedure was previously described.^9^ Briefly, hippocampi were obtained from time-mated embryonic day 16 (E16) mice and killed by CO_2_ asphyxiation. Hippocampal tissue from individual embryos was mechanically triturated in Neurobasal medium containing B27 (Invitrogen, Grand Island, NY, USA) and glutamine and plated in two 35 mm poly-D-lysine-coated petri dishes at ~350 000 cells/dish (1.5 ml medium/dish). Cultures were maintained in Neurobasal medium at 37 °C in a 95% air/5% CO_2_ humidified incubator and contained virtually pure neurons. Tail samples from individual embryos were processed for genotyping.

### Apoptosis assays

The cells (CHO cells transiently transfected as described above or primary hippocampal neurons) were washed with PBS and exposed to 1 mM H_2_O_2_ for 5 min. The cells were washed two times with PBS and incubated for 6 h in fresh medium. Then the cells were washed with PBS and incubated for 15 min with Annexin-V labeling buffer solution (20 *μ*l of Annexin-V-Fluos and 20 *μ*l of propidium iodide in 1 ml of incubation buffer provided by the kit. Propidium iodide detects both necrotic and apoptotic cells. The number of cells dying by necrosis varied between 3 and 7% in average) according to kit's instructions (Annexin-V-FLUOS staining kit no. 11858777001, Sigma). Cells were mounted on a Leica DMIRB inverted microscope (Leica, Buffalo Grove, IL, USA) equipped with a digital camera and photographed (6–8 images/culture) for subsequent analysis.

Experiments were performed blind. The fluorescence of Annexin-V in each image was calculated using the ImageJ software (NIH).

### Dose–response curve fitting

Dose–response curves were fitted to a sigmoidal function:


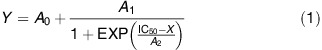


where *Y* is the fraction of apoptotic cells normalized to give 1 in the presence of H_2_O_2_ alone, *A*_0_, *A*_1_ and *A*_2_ are constants and *X* is the concentration of inhibitor expressed in nM.

### Statistical analysis

Quantitative data are presented as mean±S.E.M. All experiments were repeated in triplicate if not otherwise stated. The level of significance, assumed at the 95% confidence limit or greater (*P*<0.05), was calculated using one-way ANOVA with a Tukey's *post hoc* test (http://astatsa.com/OneWay_Anova_with_TukeyHSD).
