# Correction: Oxidation of KCNB1 potassium channels triggers apoptotic integrin signaling in the brain

**DOI:** 10.1038/s41419-019-1982-6

**Published:** 2019-10-07

**Authors:** Wei Yu, Manasa Gowda, Yashsavi Sharad, Surindo A. Singh, Federico Sesti

**Affiliations:** Department of Neuroscience and Cell Biology, Rutgers University, Robert Wood Johnson Medical School, 683 Hoes Lane West, Piscataway, NJ USA

**Correction to: Cell Death & Disease**

10.1038/cddis.2017.160, published online 6 April 2017

Since the publication of this article the authors have noted that there were two typos that could have caused confusion to the readers:

1. References to “integrin alpha chain V” should have been “integrin alpha 5”. These appear in the Abstract and the first paragraph of the Results section.
2. In the Results section under the header “Integrins activate Fyn tyrosine kinases” and the legend of Figure 6a, “tyr530” should be replaced with “tyr416”.
3. In the the legend of Figure 6b, "pSrc" should be replaced with "pFyn".

The corrected text is supplied below. These typos do not impact the scientific integrity of the article and we apologize for any inconvenience this may have caused the readers.

**Abstract**

Oxidative modification of the voltage-gated potassium (K^+^) channel KCNB1 promotes apoptosis in the neurons of cortex and hippocampus through a signaling pathway mediated by Src tyrosine kinases. How oxidation of the channel is transduced into Src recruitment and activation, however, was not known. Here we show that the apoptotic signal originates from integrins, which form macromolecular complexes with KCNB1 channels. The initial stimulus is transduced to Fyn and possibly other Src family members by focal adhesion kinase (FAK). Thus KCNB1 and integrin alpha 5 (integrin-*α*_5_) coimmunoprecipitated in the mouse brain and these interactions were retained upon channel’s oxidation. Pharmacological inhibition of integrin signaling or FAK suppressed apoptosis induced by oxidation of KCNB1, as well as FAK and Src/Fyn activation. Most importantly, the activation of the integrin–FAK–Src/Fyn cascade was negligible in the presence of non-oxidizable C73A KCNB1 mutant channels, even though they normally interacted with integrin-α_5_. This leads us to conclude that the transition between the non-oxidized and oxidized state of KCNB1 activates integrin signaling. KCNB1 oxidation may favor integrin clustering, thereby facilitating the recruitment and activation of FAK and Src/Fyn kinases.

**Integrins form stable complexes with KCNB1 channels in CHO cells and brain**

To determine whether integrins and KCNB1 channels can interact and whether these associations are retained following oxidation of the channel, we carried out coimmunoprecipitation experiments in Chinese hamster ovary (CHO) cells transiently expressing human KCNB1 epitope tagged to the HA tag in the C-terminus (we showed previously that the addition of the tag has no effect on the properties of the channel)^8^. KCNB1 was oxidized by exposing the cells to 1.0 mM hydrogen peroxide, (H_2_O_2_) for 5 min prior to lysis. Proteins were immunoprecipitated (IP) with an antibody that detects integrins alpha 5 (integrin-α5) and immunoblotted with a HA antibody to detect KCNB1 protein. As a control, we used a KCNB1 mutant, C73A, which does not oligomerize^8^. Representative western blots of six coimmunoprecipitation experiments are shown in Figure 1. The lower blot in Figure 1a shows staining of total lysates with integrin-α5 antibody. Coimmunoprecipitations are shown in the upper blot in the figure. Oxidized KCNB1 channels in CHO cells or in the mouse brain form oligomers that can be detected in multiple bands ranging from ∼170 to ∼400 kDa.^8^ Indeed, integrin-α5 pulled down both the non-oxidized (∼110 kDa) and oxidized (∼200 kDa) forms of wild-type (WT) KCNB1 indicating that the channel interacted with endogenous integrins in CHO cells and that these interactions were retained upon its oxidation/oligomerization. Also C73A channels formed a complex with the integrins but in this case only the non-oxidized band was detected in the blot because C73A does not oligomerize.

**Figure 6 legend**

Cyclo and PND-1186 inhibit Fyn activation induced by oxidation of KCNB1. **a** Representative western blots showing phosphorylated Fyn at tyr416 (pFyn) and total Fyn (Fyn) in CHO cells transfected with WT or C73A in the absence/presence of the indicated inhibitors. Fyn protein was detected into a single, ∼60 kDa band. Cells were oxidized with 1.0 mM H_2_O_2_ for 5 min and then incubated in control media or in media containing 200 nM Cyclo or 10 nM PND-1186 (PND) for 1 h before lysis. Quantifications of three experiments are shown in the lower panel and are normalized to WT+H_2_O_2_. *P* = 1.3 × 10^−6^ and 7.7 × 10^−7^ for Cyclo and PND-1186, respectively (one-way analysis of variance (ANOVA)). **P < 0.01 for pairwise comparisons *versus* WT+H_2_O_2_ (Tukey’s post hoc test). **b** Representative western blots showing phosphorylated Fyn (pFyn) and total Fyn (Fyn) in the brains of the indicated genotypes in the absence/presence of Cyclo or PND-1186. Brain lysates were incubated 1 h in the absence/presence of 1.0 mM H_2_O_2_ and the absence/presence of 200 nM Cyclo or 10 nM PND-1186 (PND). The reactions were stopped by adding sample buffer to the lysates. Quantifications of three experiments with non-Tg and Tg-C73A are shown in the lower panel and are normalized to non-Tg+H_2_O_2_. *P* = 9.8 × 10^−8^ (one-way ANOVA). **P < 0.01 for pairwise comparisons versus non-Tg+H_2_O_2_ (Tukey’s post hoc test). Quantifications of two experiments with Tg-WT are shown in the lower panel and are normalized to Tg-WT+H_2_O_2_. *P* = 1.5 × 10^−4^ (one-way ANOVA). **P < 0.01 for pairwise comparisons versus Tg-WT+H_2_O_2_ (Tukey’s post hoc test).

**Integrins activate Fyn tyrosine kinases**

We next determined whether FAK was responsible for the recruitment and activation of Src kinases that follows oxidation of KCNB1. To assess the fraction of activated Src kinases, we used an antibody that detects phosphorylation status of tyr416, a residue conserved in all members of the Src kinase family, as carried out before^9^^,^^24^. Thus, in CHO cells transfected with WT or C73A, Src phosphorylation at tyr416 was negligible at baseline (Fig. 5a). Following an oxidative insult, Src phosphorylation was significantly increased in cells expressing the WT channel compared with cells expressing the C73A mutant, and most importantly, treatments with 200 nM Cyclo or 10 nM PDN-1186 suppressed it. Similar results were observed in the brains of non-Tg, Tg-WT and Tg-C73A mice, with the amounts of phosphorylated Src ∼32% larger in the Tg-WT brains compared with the non-Tg brains (Fig. 5b. In the figure, data are plotted normalized to their internal control). The family of Src tyrosine kinases is composed of nine members, including Fyn, which belongs to the SercA sub-family and has been implicated in TBI and Alzheimer’s disease, two conditions associated with robust KCNB1 oxidation^8^^,^^9^^,^^17^^,^^25^^,^^26^. Therefore, we next sought to determine whether oxidation of KCNB1 resulted in the activation of Fyn using a specific antibody that recognizes phosphorylated tyr416 in Fyn. Figure 6a shows the fraction of phosphorylated Fyn kinases in CHO cells transfected with WT or C73A and Fig. 6b in the brain lysates of non-Tg, Tg-WT and Tg-C73A in control conditions or following exposure to 1.0 mM H_2_O_2_. The fraction of phosphorylated Fyn protein at tyr416 following an oxidative challenge was significantly increased in all cells expressing the WT channel compared with control and remained low in cells expressing the C73A mutant. Further, Fyn phosphorylation was significantly decreased by treatment with Cyclo or PND-1186.

